# *NAT2* Gene Polymorphisms in Turkish Patients with Psoriasis Vulgaris

**DOI:** 10.1155/2018/3258708

**Published:** 2018-06-11

**Authors:** Recep Dursun, Hatice Gül Dursun, Ayşe Gül Zamani, Mahmut Selman Yıldırım, İlknur Çınar

**Affiliations:** ^1^Department of Dermatology, Meram Faculty of Medicine, Necmettin Erbakan University, 42080 Konya, Turkey; ^2^Department of Medical Biology, Meram Faculty of Medicine, Necmettin Erbakan University, 42080 Konya, Turkey; ^3^Department of Medical Genetics, Meram Faculty of Medicine, Necmettin Erbakan University, 42080 Konya, Turkey

## Abstract

Psoriasis is a common, chronic, and autoimmune skin disease. Factors that play a role in etiopathogenesis of psoriasis include internal factors such as genetic susceptibility and immunological factors and external factors such as stress, infection, trauma, drug, and environmental compounds. N-acetyltransferase 2 (NAT2) is a xenobiotic enzyme that is involved in the metabolism of drugs, environmental toxins, and carcinogens. In this study, we aimed to demonstrate whether the variations in the* NAT2* gene lead to a predisposition to psoriasis by affecting the enzyme's ability to metabolize drugs and environmental components or not. Three polymorphisms (*rs1799929, rs1799930*, and* rs1799931*) in* NAT2* gene were genotyped and compared by real-time PCR method in 260 psoriasis vulgaris patients and 200 healthy controls. There was no difference in the genotype distributions and allele frequencies of polymorphisms between psoriasis vulgaris patients and controls. When the effects of polymorphisms on the clinical features of the disease, such as onset age and severity, are assessed, it has been found that* rs1799930* and* rs1799929 *are, respectively, associated with early onset age and severity of the disease. In conclusion,* rs1799929, rs1799930*, and* rs1799931* polymorphisms of the NAT-2 gene do not appear to be a risk factor for the development of psoriasis. Conversely, they may have an effect on either more severe or early onset cases of the disease.

## 1. Introduction

Psoriasis is a common, chronic, and recurrent autoimmune inflammatory skin disease that can also affect nails and joints [1]. It is characterized by erythematous, scaly, sharply demarcated papules and plaques on skin and scalp [2]. It can be seen in all age groups without gender discrimination [1]. The prevalence of the disease is between 1% and 3% worldwide, varying among different ethnic populations and geographical regions [3]. Psoriasis has four major clinical phenotypes, which are distinguished by morphological characteristics of the associated lesions: (i) psoriasis vulgaris, (ii) guttate psoriasis, (iii) pustular psoriasis, and (iv) erythrodermic psoriasis. The most common one of these clinical phenotypes is psoriasis vulgaris, accounting for 90% of all cases, and is also known as plaque psoriasis [4]. In this phenotype, the lesions are dry, sharply demarcated, oval/circular plaques and can be localized all over the body, mostly affecting the knees, elbows, lumbosacral area, intergluteal cleft, and scalp [4, 5]. Although the etiopathogenesis of psoriasis is not fully understood, it is known that the disease manifests as a result of biochemical, immunological, and vascular system abnormalities in the presence of a genetic predisposition [6, 7]. Mutual interactions of T-cells which play a central role in pathogenetic mechanism, other immune-related cells, skin cells, and proinflammatory cytokines and chemokines cause histopathologic changes observed in psoriatic skin [8]. These histopathological changes bring out the clinical picture of the disease, which involves hyperproliferation and aberrant differentiation of keratinocytes, capillary dilatation, angiogenesis, and leukocytes infiltration into dermis [8–10]. The reported number of cellular and molecular factors contributing to its etiopathogenesis is increasing day by day [11–18]. Psoriasis is associated with several comorbidities, such as inflammatory bowel diseases [19, 20], cardiovascular syndrome [21, 22], metabolic syndrome [22–24], depression [25], and cancer [26, 27]. The disease leads to a serious reduction in patient's life quality, due to its link with social stigmatization, pain, discomfort, physical disability, aesthetic concerns about physical appearance, and psychological distress [28, 29]. Now, it is accepted that environmental factors such as trauma, infections, exposure to ultraviolet (UV) radiation, drugs, stress, and smoking/alcohol consumption play an effective role in emergence and development of psoriasis [30–33].

As in any other autoimmune disease, the pathogenesis of psoriasis requires three puzzle components to come together: genetic predisposition, environmental factors, and immune regulation disturbance [34–36]. Studies have shown that environmental factors such as trauma, infections, intestinal microbiota, exposure to UV radiation, drugs, stress, and dietary components can be the largest component of this puzzle [37, 38]. In recent years, many studies have been published on polymorphisms in genes which code xenobiotic metabolizing enzymes, in order to better understand the role of environmental factors in the pathogenesis of diseases [39–49]. One of the enzymes involved in xenobiotic metabolism is N-acetyl transferase II (NAT2). It performs detoxification of xenobiotics, which have the structure of aromatic amines, hydrazine, sulphonamides, and aliphatic amines, by acetylation reactions. These xenobiotics include environmental and dietary compounds, carcinogens, and drugs used to treat a variety of diseases, particularly tuberculosis, AIDS-related complex diseases, psoriatic arthritis, and hypertension [50–52]. NAT2 enzyme is encoded by intronless* NAT2* gene that is located at 8p22.* NAT2* gene is highly polymorphic and so far 106* NAT2* variants have been identified (http://nat.mbg.duth.gr).* NAT2* variants are often in the form of single nucleotide polymorphisms (SNP) and alter the catalytic activity or stability of the enzyme, leading to amino acid substitution [53, 54]. These alterations in gene and protein products determine the acetylation phenotypes that can be observed individually as “rapid” and “slow” acetylator phenotypes [55]. NAT2 acetylation polymorphisms are also very important in pharmacological, toxicological, and clinical aspects, since some of these polymorphisms may lead to adverse drug reactions or decreased drug effectiveness and even to increased susceptibility to certain diseases via decreasing the enzyme's acetylation capacity [51, 52].

There are a number of studies showing that* NAT2* gene polymorphisms lead to increased susceptibility to various diseases, primarily cancer [40, 56–61] and autoimmune disorders [47, 50, 62–64]. However, to the best of our knowledge, there are only two studies on the association of* NAT2* gene variants with psoriasis vulgaris [65, 66]. Only in one of these studies [65] the association of* NAT2* polymorphisms with age of the disease onset has been investigated. In our study, however, we simultaneously assessed whether* NAT2* polymorphisms were associated with the age of disease onset and the severity of the disease. The starting point of our study is the two fundamental principles about psoriasis and NAT2: (1) psoriasis pathogenesis can be triggered by drugs and various other environmental factors. (2) NAT2 enzyme is responsible for detoxification of various drugs and environmental compounds. In that case, can* NAT2* gene acetylation polymorphisms contribute to the pathogenesis of the disease by facilitating the factors that may trigger psoriasis? With this hypothesis, our goals were (i) to investigate whether the* NAT2* gene polymorphisms are related to development of psoriasis vulgaris (ii) and to detect whether the* NAT2* gene polymorphisms have an impact on the clinical features of psoriasis vulgaris such as age of onset and severity.

In the present study, we conducted a research on 481C>T (rs1799929), 590G>A (rs1799930), and 857G>A (rs1799931) in the* NAT2* gene. We also confirmed that the acetylator phenotypes are induced by these three SNPs at “http://nat.mbg.duth.gr”: rs1799929 is responsible for the rapid acetylator phenotype while rs1799930 and rs1799931 are responsible for the slow acetylator phenotype. We chose these SNPs, as they are among the most commonly used SNPs for inferring NAT2 acetylator phenotype [64], and rs*1799930* and rs*1799931* especially are defined to be the cause of 95% of the low enzymatic activity alleles [67].

## 2. Materials and Methods

### 2.1. Subjects

Two hundred and sixty (260) unrelated Turkish psoriasis vulgaris patients and two hundred (200) unrelated Turkish healthy controls were included in the study. Psoriasis vulgaris patients (142 females/118 males; mean age±SD: 39.83± 24.52) were recruited from the Dermatology Clinic. The patients with other chronic and autoimmune disorders or cancer were excluded from the study. The control subjects (99 females/101 males; mean age±SD: 38.94±18.80) were healthy individuals who had been examined for psoriasis, cancer, autoimmune symptoms, and family history. The patients and controls were matched according to their gender and age. Severity of psoriasis was assessed via Psoriasis Area and Severity Index (PASI), ranging from 0 (no disease) to 72, with higher scores indicating the severity of disease [68]. To determine the level of association of* NAT-2* gene polymorphisms with clinical features of psoriasis vulgaris, the patients were divided into two groups according to the severity of disease (PASI<12 group and PASI≥12 group) and then assigned into two groups according to the onset of disease (early onset group: <40 age; late onset group: ≥40 age) (Table 1). This study was conducted in accordance with the Declaration of Helsinki principles and was approved by the Meram Medical Faculty Ethics Committee (No: 2011/204). Informed consent was obtained from all participants before beginning of the study.

### 2.2. Genotyping

Blood specimens from psoriasis vulgaris patients and controls were drawn into EDTA-containing tubes and were kept at 4°C until the blood collection from all subjects was completed. Genomic DNA from peripheral blood leukocytes of all patients and controls was extracted with the High Pure PCR Template Preparation Kit (Roche Molecular Diagnostics, Manheim, Germany) according to the manufacturer's instructions.

Genotyping of* rs1799929* (481C>T),* rs1799930* (590G>A), and* rs1799931* (857G>A) was performed by using the LightCycler-NAT2 mutation detection kit by real-time PCR with the LightCycler 480 instrument (Roche Molecular Diagnostic, Manheim, Germany). Reaction mixture for real-time PCR comprised 10.4-14.4 *μ*l distilled water, 1.6 *μ*l MgCl_2_ (25 mM), 10 pmol of each primer, 4 pmol of each probe, and 2 *μ*l genomic DNA (50-200 ng/ml). The cycling conditions were as follows: an initial denaturation step at 95°C for 10 min followed by 45 cycles for 10 s at 95°C, 10 s at 62°C, and 15 s at 72°C. Wild type, heterozygote, and polymorphic genotypes were detected by specific melting temperature (Tm) of amplicons.

### 2.3. Statistical Analysis

The expected genotype values for testing Hardy-Weinberg equilibrium (HWE) were calculated from allele frequencies. Deviations from observed genotype values were determined by *χ*^2^-test. The SNPStats program was used to test the relationship between genotype and disease according to different inheritance models. Haploview version 4.2 was also applied to construct haplotype structures and to determine the relationship between haplotype and disease.

## 3. Results

### 3.1. The Characteristics of Patients and Controls

**Figure 1:**
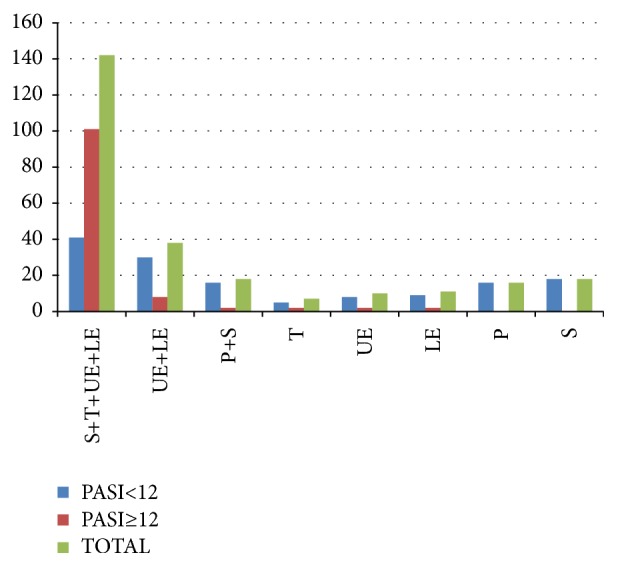
The diagrammatic distribution of psoriatic lesions in two PASI groups.** S:** scalp,** T:** trunk,** UE:** upper extremities,** LE:** lower extremities, and** P:** palmoplantar.

The general characteristics of patients and controls included in the study are presented in Table 1. The study included 260 psoriasis patients (118 males, 142 females) and 200 healthy individuals (101 males, 99 females). The mean age of the subjects was 39.83 ± 24.52 years for patients and 38.94 ± 18.8 years for controls. The distributions of gender and age were not significantly different between cases and controls. The patients were divided into subgroups according to onset age of psoriasis vulgaris (early onset group: <40 age; late onset group: ≥40 age) and severity of psoriasis vulgaris (PASI<12 group; PASI≥12 group) (Figure 1).

### 3.2. Association of NAT2 Gene Polymorphisms with the Risk of Developing Psoriasis Vulgaris

#### 3.2.1. Genotype and Allele Frequency Distribution

The genotype and allele frequencies of studied* NAT2* gene polymorphisms in psoriasis vulgaris patients and controls are shown in Table 2. The genotypic distributions of SNPS were consistent with the Hardy-Weinberg equilibrium (HWE). The p values for the HWE of* rs1799929*,* rs1799930*, and* rs1799931* were 0.11/0.33 (patient/control), 0.53/0.07, and 0.47/0.63, respectively. NAT2 genotypes were compared according to four different inheritance patterns and allele frequencies in patients and controls and no significant differences were found.

#### 3.2.2. Acetylator Phenotype Distribution

Table 3 shows the distribution of expected acetylation phenotypes in psoriasis vulgaris patients and controls. The fast acetylator phenotype frequency expected from TT/GG/GG genotypes was significantly higher in psoriasis vulgaris patients (OR=2.33, 95% CI=1.06-5.12, and p=0.03). However the total frequency of slow acetylator phenotype was not significantly higher in patients when compared with controls (OR=0.78, 95% CI=0.54-1.13, and p=0.19).

#### 3.2.3. Haplotype Analysis

**Figure 2:**
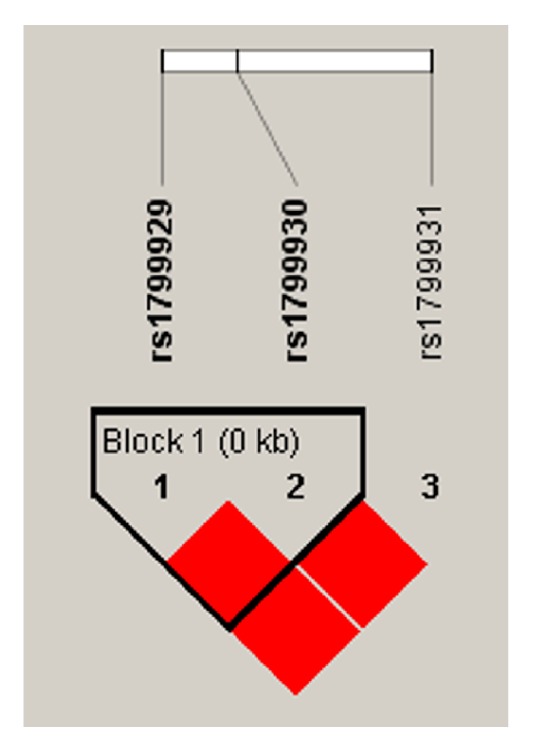
Linkage disequilibrium (LD) map of* NAT2* gene polymorphisms.

Linkage disequilibrium (LD) map of SNPs was genotyped using Haploview (Figure 2).* rs1799929* and* rs1799930* were in strong LD and form a haplotype block.* rs1799931* located outside of this block was not in LD with* rs1799929* and* rs1799930*. Haplotype analysis was performed using all 3 SNPs (*rs1799929*,* rs1799930*, and* rs1799931*) and 2 SNPs (*rs1799929* and rs*1799930*) at the same block. Four haplotypes were detected in patients and controls among which the frequencies did not differ significantly (Table 4).

### 3.3. Association of NAT2 Gene Polymorphisms with Clinical Characteristics of Psoriasis Vulgaris

Firstly,* NAT2 *polymorphisms were evaluated for having an effect on the onset age of psoriasis vulgaris. The comparisons of genotypes with the onset age of psoriasis vulgaris under the genetic models were presented in Table 5. We observed an increased risk of early onset age of psoriasis vulgaris in* rs1799930* according to the codominant model (OR=1.75, 95% CI=0.65-4.73, and p=0.046, for the AA genotype) and to the overdominant model (OR=0.44, 95% CI=0.21-0.93, and p=0.026, for the GA genotype).

The distribution of acetylator phenotypes in early onset age and late onset age groups was shown in Table 6. There was no significant difference in the distribution of the slow acetylator and fast acetylator phenotypes between the early onset and late onset age groups.

Haplotype analysis results were presented in Table 7. None of the haplotypes had a significant relationship with the onset age of psoriasis vulgaris.

Secondly, we assessed whether* NAT2* polymorphisms were associated with the severity of psoriasis vulgaris (Table 8). We observed an increased risk of severity (PASI≥12) of psoriasis vulgaris in* rs1799929* according to the dominant model (OR=0.57, 95% CI=0.35-0.94, and p=0.026, for the CC+TT genotypes). The minor allele (T) frequency was also significantly higher in the PASI≥12 (severe) group compared to the PASI<12 (mild) group (OR=0.68, 95% CI=0.47-0.98, and p=0.04).

The distribution of acetylator phenotypes in PASI≥12 and PASI<12 groups is given in Table 9. Although the frequencies of TT/GG/GG-fast acetylator, TT/GA/GA-slow acetylator, and CT/GA/GA-slow acetylator phenotypes were found to be significantly higher in the PASI≥12 group (p=0.04, p=0.04, and p=0.03, respectively), total fast and total slow acetylator phenotype frequencies did not differ significantly between the PASI≥12 and PASI<12 groups.

In haplotype analysis, no significant difference in haplotype distribution was detected in the PASI≥12 group compared to the PASI<12 group (Table 10).

## 4. Discussion

In recent years, scientists have turned their attention to polymorphisms in genes encoding xenobiotic enzymes to elicit the effect of gene-environment interaction on multifactorial diseases such as cancer, neurological diseases, and autoimmune diseases [39–49]. One of the enzymes involved in xenobiotic metabolism is NAT2. NAT2 is a phase II enzyme that catalyzes the acetylation reactions to perform the detoxification of xenobiotics, including environmental/dietary compounds, carcinogens, and drugs [50–52].

The* NAT2* gene, which encodes the NAT2 enzyme, is a highly polymorphic gene. The polymorphisms of the* NAT2* gene have major pharmacogenetic considerations as they lead to adverse drug reactions and individual differences in drug response [51, 52].* NAT2* polymorphisms have also clinical importance, due to the fact that these polymorphisms lead to susceptibility to many diseases such as autoimmune diseases and cancer, for which the etiopathogenesis is affected by environmental factors [40, 47, 50, 56–64]. Indeed, psoriasis is one of these diseases with an etiopathogenesis affected by environmental factors.

In the present study, we investigated whether the most common three polymorphisms (481C>T, 590G>A, and 857G>A), effective in the acetylation phenotype of the* NAT2* gene, have any effect on the development and clinical features of psoriasis vulgaris, i.e., the most common clinical type of psoriasis. The study was performed with 260 patients (118 males/142 females) and 200 controls (101 males/99 females). Although there was no statistically significant difference in sex ratios between the two groups, the proportion of females (54.62%) was slightly higher than that of males (45.38%). Despite various studies reporting that the incidence of psoriasis is higher either in females or males [33], there is a theoretical consensus on no gender discrimination in psoriasis incidence [6]. In a study on the incidence of psoriasis in Turkey, the prevalence of the disease is given as 1.2% in females and as 1.0% in males [69]. In another study, the female/male ratio in Turkish psoriasis patients has been reported as 200/129 [70]. In our study, it is possible to explain the excess of women in the group of psoriasis as follows: psoriatic women are more often referred to clinic than psoriatic men, due to their physical appearance/aesthetic concerns.

In our study, the genotype and allele frequencies of three polymorphisms of the* NAT2* gene (481C>T, 590G>A, and 857G>A) were compared between psoriasis vulgaris patients and controls. The genotypes and allele distributions of three polymorphism did not manifest a statistically significant difference between patients and controls, according to genetic models we applied. No significant association was observed between acetylator phenotypes and susceptibility to psoriasis vulgaris either. To our knowledge, there are only two studies on the relationship between psoriasis and* NAT2* polymorphisms in the literature. In one of these studies, Reich et al. [65] studied seven* NAT2* polymorphisms, including 481C>T, 590G>A, and 807G>A, in 151 psoriasis patients and 123 controls. They found no significant differences in the distribution of* NAT2* polymorphisms between psoriasis patients and controls. In another study, Kozhekbaeva et al. [66] studied* NAT2* 282C>T, 341T>C, 481C>T, 590G>A, 803A>G, and 857G>A polymorphisms in 180 psoriasis patients and 99 controls, with the findings that suggest 341T>C, 481C>T, and 803A>G polymorphisms and some lifestyles could be considered as risk factors of psoriasis development.

There is a great deal of research on the possible association of* NAT2* gene polymorphisms with other autoimmune diseases. The results of studies on inflammatory bowel disease (IBD) are contradictory. Baranska et al. [63] evaluated 481C>T, 803A>G, 590G> A, and 857G>A polymorphisms in IBD and found that only the 857A allele was correlated with IBD. Similarly, in another study on IBD patient group, it was reported that* NAT2∗*6A (282C>T, 590G>A) haplotype had correlation with neither of ulcerative colitis and Crohn's disease, with a higher frequency of* NAT2∗*7B (282C>T, 857G>A) haplotype found only for Crohn's disease [71]. Chen et al. [72] have studied the same polymorphisms as those in our study, with consistent results. The researchers found that 481C>T, 590G>A, and 857G>A polymorphisms did not count as a susceptibility factor for inflammatory bowel disease but that slow acetylator genotypes were significantly associated with dose-related adverse effects of sulfasalazine in the treatment of inflammatory bowel disease.

The more frequent occurrence of drug-induced systemic lupus erythematosus (SLE) in individuals with slow acetylator genotypes has led to hypothesis that xenobiotics accumulate and convert into reactive molecules due to defect in acetylation to stimulate immune system and T-cells in favor autoimmunity [73]. However, in idiopathic SLE, the role of the acetylator genotype and phenotypes on susceptibility to disease is controversial. The study on the association of* NAT2* polymorphisms with SLE overall shows that 481C>T, 590G>A, and 857G>A polymorphisms and slow acetylator genotypes do not form a risk factor for SLE [74]. But Lima dos Santos et al. [64] reported that only 857G>A polymorphism among 481C>T, 590G>A, 857G>A, and 191G>A polymorphisms was associated with SLE.

In a study conducted with a very small systemic sclerosis patient population (n=39), the distributions of 481C>T, 590G>A, 803A>G, and 857G>A polymorphisms have been compared between patients and controls and no significant difference was found [75].

*NAT2* acetylation polymorphisms have also been studied in rheumatoid arthritis (RA). In a study evaluating* NAT2∗*4,* NAT2∗*5,* NAT2∗*6, and* NAT2∗*7 genotypes, it has been suggested that* NAT2* slow acetylator genotypes might lead to RA susceptibility [62]. Oqal et al. [76] also have studied the same polymorphisms and found that only the* NAT2 ∗*5/7 genotype correlates with RA, while other slower acetylator genotypes are not a risk factor for RA.

In the case-control study conducted by Tamer et al. it was suggested that* NAT2∗*5A and* NAT2∗*6A slow acetylator genotypes were significantly associated with Behçet's disease [47].

In the current study, we also aimed to assess whether the* NAT2* polymorphisms have any effect on the clinical characteristics of psoriasis (such as the age of the disease onset and the severity of the disease). We compared the distribution of genotype and allele frequencies of polymorphisms between “early onset age” and “late onset age” and between “PASI<12” and “PASI≥12” groups. In the analyses, we found a statistically significant correlation between the 590G>A polymorphism and the age of the disease onset according to the codominant (p=0.046) and overdominant (p=0.026) inheritance patterns; however the two other polymorphisms did not correlate with age of onset. It is not surprising that we found a relationship between 590G>A polymorphism and age of the disease onset according to codominant and overdominant inheritance patterns. As a matter of fact, the comparisons were made according to the 4 inheritance patterns and allele frequencies. The alleles frequencies did not differ between the two groups while a correlation was found according to the two of inheritance patterns. Given the statistically significant difference between two groups according to only two inheritance patterns, suggesting 590G> A polymorphism to be a distinct risk factor for the early age onset of psoriasis would not be realistic.

In the analysis of polymorphisms-disease severity relations, we only found the frequency of CT+TT genotypes (p=0.026) and minor allele (T allele) frequencies of 481C>T polymorphism significantly higher in PASI≥12 group compared to PASI<12 group. Other polymorphisms did not correlate with the severity of the disease. Concordantly, 481C>T polymorphism being associated with the severity of the disease is not surprising, according to only the dominant inheritance pattern. However, the fact that the frequency of the minor allele (T) was higher in the PASI≥12 group than in the PASI<12 group suggests that 481C>T polymorphism may have a precautionary effect on the severity of the disease. The association of* NAT2* polymorphisms with the clinical features of psoriasis has only been analyzed by Reich et al. [65]. The researchers evaluated the association of* NAT2* polymorphisms with age of the disease onset. Their data suggest a relationship between early onset age (<40 age) and fast acetylator phenotype. When we consider the acetylator phenotypes in our study, we found no relationship between the acetylator phenotypes and the age of the disease onset. When we evaluated the relationship between the severity of the disease and acetylator phenotypes, TT/GG/GG-fast acetylators (p=0.04), TT/GA/GA-slow acetylators (p=0.04), and CT/GA/GA-slow acetylators (p=0.03) were all statistically significantly higher in PASI≥12 group compared to the PASI <12 group (order of genotypes is* rs1799929*/*rs1799930*/*rs1799931*).

Here, the question might come to mind: Could the relationship between certain SNPs and age of onset/severity of the disease be due to linkage disequilibrium between these alleles and functional polymorphisms at distant sites? However, to the best of our knowledge, none of the polymorphisms directly associated with psoriasis are found on chromosome 8p [77, 78]. We, therefore, think that the relationship between certain SNPs and the clinical features of the psoriasis vulgaris is directly related to the NAT2 itself, rather than to linkage disequilibrium between these alleles and functional polymorphisms.

Our study has some limitations: (i) We evaluated the polymorphisms of gene encoding NAT2 enzyme that work in the detoxification of environmental compounds and drugs in psoriasis patients, but we could not question or categorize lifestyle, nutritional habits, drug use, smoking-alcohol habits, and environmental/occupational exposures of patients and controls. (ii) It was not possible for us to analyze all known polymorphisms of* NAT2* gene. These two issues make it difficult to assess a disease affected by the gene-gene and/or gene-environment interactions during etiopathogenesis, such as psoriasis.

*NAT2* is extensively polymorphic, and the distribution of polymorphic alleles varies widely between populations and ethnic groups.  Table 11 shows the distribution of allele frequencies of the* NAT2* polymorphisms* rs1799929*,* rs1799930*, and* rs1799931* in healthy individuals from different ethnic groups. The allele distributions of* rs1799929*,* rs1799930*, and* rs1799931* in our study population are close to the respective distribution of Indian [84] and Mexican [82], British [95] and Polish [92], and Egyptian [79] and Polish [92] populations.

In a study carried out by Tamer and colleagues [94] for the Turkish population living in Mersin (a city in South Anatolia), polymorphic allele frequencies have been reported as 0.34 for* rs1799929*, 0.23 for* rs1799930*, and 0.23 for* rs1799931*. Our study was conducted on the other hand in Konya (in central Anatolian city) and the results we found were similar to Tamer and colleagues' study population only in terms of* rs1799929* allele frequencies. The distributions of* rs1799930* and* rs1799931* allele frequencies differ between the two populations. Presumably these differences stem from the ethnic differences of the populations in the two major cities of Anatolia. This suggests that the* NAT2* gene is so polymorphic that it can cause serious differences in the allele frequency even between populations of two neighboring cities in the same geographical region.

## 5. Conclusions

According to the data of this study,* NAT2* gene 481C>T, 590G>A, and 807G>A polymorphisms do not appear to be effective in the emergence of psoriasis vulgaris disease. However, further studies should consider the possibility that 590G> A polymorphism may have an effect on the age of the disease onset and 481C> T polymorphism may have an effect on the severity of the disease. As a result, the* NAT2* is a very remarkable gene due to its high polymorphism rates and the crucial role of the enzyme it encodes in several detoxification events. This high polymorphism rate and variation variability also necessitate working with large populations and as many relevant individuals/cases as possible in studies.* NAT2* will continue to be a suspicious gene in the pathogenesis of many diseases due to the role of the enzyme it encodes. Even though our findings indicated that* NAT2* polymorphisms are not directly related to the pathogenesis of psoriasis, these polymorphisms may play a role in the age of onset and severity of the disease. In this regard, more detailed studies should be carried out in larger populations to demonstrate the relationship between* NAT2* gene polymorphisms and psoriasis.

## Figures and Tables

**Table 1:** Clinical characteristics of study subjects.

	**Case**	**Control**	
	**n=260**	**n=200**	***P***
**Age (years)**			
Mean (±SD)	39.83±24.52	38.94±18.8	0.67
**Gender**			
Male	118 (45.38)	101 (50.5)	0.30
Female	142 (54.62)	99 (49.5)	
**Onset age of psoriasis**			
Early onset (<40 age)	220 (84.62)		
Late onset (≥40 age)	40 (15.38)		
**Severity of psoriasis**			
PASI≥12 (severe)	117 (45)		
PASI<12 (mild)	143 (55)		
**Family history of psoriasis**	No (-)	No (-)	
**Accompanying disease**			
Cancer	No (-)	No (-)	
Autoimmune disease	No (-)	No (-)	
Systemic disease	No (-)	No (-)	
Psoriatic arthritis	No (-)	No (-)	

**Table 2:** Genotype and allele frequencies of *NAT2* gene polymorphisms in psoriasis vulgaris patients and controls and association of these polymorphisms with psoriasis vulgaris risk.

**SNP**	**Model**	**Genotype/** **Allele**	**Cases** **n(%)**	**Controls** **n(%)**	**HWE p** **(patient)**	**HWE p** **(control)**	**OR (95% CI)**	**p**
***NAT2*** **∗** **11A** rs1799929 (481C>T)	Codominant	CC	119 (45.7)	87 (43.5)	0.11	0.33	Ref.	0.88
		CT	105 (40.4)	85 (42.5)			1.11 (0.74-1.65)	
		TT	36 (13.8)	28 (14.0)			1.06 (0.60-1.87)	
	Dominant	CC	119 (45.7)	87 (43.5)			Ref.	0.63
		CT+TT	141 (54.2)	113 (56.5)			1.10 (0.76-1.59)	
	Recessive	CC+CT	224 (86.2)	172 (86)			Ref.	0.96
		TT	36 (13.8)	28 (14)			1.01 (0.59-1.72)	
	Overdominant	CC+TT	155 (59.6)	115 (57.5)			Ref.	0.65
		CT	105 (40.4)	85 (42.5)			1.09 (0.75-1.59)	
	Major Allele	C	343 (65.9)	259 (64.8)			Ref.	0.70
	Minor Allele	T	177 (34.0)	141 (35.2)			1.05 (0.80-1.39)	

***NAT2*** **∗** **6B** rs1799930 (590G>A)	Codominant	GG	122 (46.9)	82 (41)	0.53	0.07	Ref.	0.32
		GA	109 (41.9)	101 (50.5)			1.33 (0.90-1.96)	
		AA	29 (11.2)	17 (8.5)			1.01 (0.51-1.99)	
	Dominant	GG	122 (46.9)	82 (41)			Ref.	0.2
		GA+AA	138 (53.1)	118 (59)			1.27 (0.88-1.85)	
	Recessive	GG+GA	231(88.8)	183 (91.5)			Ref.	0.68
		AA	29 (11.2)	17 (8.5)			0.87 (0.46-1.67)	
	Overdominant	GG+AA	151 (58.1)	99 (49.5)			Ref.	0.13
		GA	109 (41.9)	101 (50.5)			1.33 (0.92-1.92)	
	Major Allele	G	353 (67.9)	265 (66.9)			Ref.	0.60
	Minor Allele	A	167 (32.1)	135 (33.7)			1.08 (0.82-1.42)	

***NAT2*** **∗** **7A** rs1799931 (857G>A)	Codominant	GG	237 (91.2)	187 (93.5)	0.47	0.63	Ref.	0.46
		GA	22 (8.4)	13 (6.5)			0.79 (0.38-1.61)	
		AA	1 (0.4)	0			0.00	
	Dominant	GG	237 (91.2)	187 (93.5)			Ref.	0.433
		GA+AA	23 (8.8)	13 (6.5)			0.75 (037-1.53)	
	Recessive	GG+GA	259 (99.6)	200 (100)			Ref.	0.29
		AA	1 (0.4)	0			0.00	
	Overdominant	GG+AA	238 (91.5)	187 (93.5)			Ref.	0.52
		GA	22 (8.4)	13 (6.5)			0.79 (0.39-1.62)	
	Major Allele	G	495 (95.6)	387 (96.8)			Ref.	0.29
	Minor Allele	A	23 (4.4)	13 (3.2)			1.44 (0.72-2.87)	

*∗*SNP: single nucleotide polymorphism; HWE: Hardy-Weinberg equilibrium; OR: odds ratio; CI: confidence interval.

**Table 3:** Distribution of expected acetylation phenotypes in patients and controls.

**rs1799929**	**rs1799930**	**rs1799931**	**Phenotype**	**Cases** **n (%)**	**Controls** **n (%)**	**Total** **n (%)**	**OR** **(95% CI)**	**p**
CC	GG	GG	Fast	18 (6.92)	21 (10.50)	38 (8.26)	Ref.	
TT			Fast	52 (20)	26 (13)	78 (16.96)	2.33 (1.06-5.12)	**0.03**
	GA		Fast	52 (20)	39 (19.50)	91 (19.78)	1.55 (0.73-3.31)	0.25
		GA	Fast	11 (4.23)	4 (2)	15 (3.26)	3.21 (0.87-11.8)	0.08
TT			Slow	36 (13.85)	28 (14)	64 (13.91)	1.50 (0.67-3.34)	0.32
	AA		Slow	29 (11.15)	17 (8.50)	46 (10)	1.99 (0.83-4.74)	0.12
		AA	Slow	1 (0.38)	0	1 (0.22)	-	-
	AA	GA	Slow	0	0	0	-	-
CT	GA		Slow	49 (18.85)	56 (28)	105 (22.83)	1.02 (0.49-2.13)	0.95
CT		GA	Slow	4 (1.54)	3 (1.50)	7 (1.52)	1.55 (0.31-7.89)	0.59
	GA	GA	Slow	8(3.08)	6 (3)	14 (3.04)	3.11 (0.72-13.51)	0.13

**Total Fast n (%)**				133 (51.15)	90 (45)	223 (48.48)	Ref	
**Total Slow n (%)**				127 (48.85)	110 (55)	237(51.52)	0.78 (0.54-1.13)	0.19

*∗*OR: odds ratio; CI: confidence interval.

*∗*Boldface indicates statistical significance.

**Table 4:** Distribution of haplotype frequencies in patients and controls and association of these haplotypes with psoriasis vulgaris.

**Haplotype**	**Case (f)**	**Control (f)**	**OR (95%CI)**	**p**
TGG	0.3404	0.3525	Ref.	
CAG	0.3235	0.3375	1.06 (0.77-1.46)	0.73
CGG	0.3019	0.2775	0.90 (0.65-1.24)	0.51
CGA	0.0442	0.0325	0.73 (0.36-1.47)	0.38
TAG	0	0	0	-
CAA	0	0	0	-

*∗*OR: odds ratio; CI: confidence interval.

**Table 5:** Genotype and allele frequencies of *NAT2* gene polymorphisms in early onset group and late onset group and association of these polymorphisms with onset of psoriasis vulgaris.

**SNP**	**Model**	**Genotype/** **Allele**	**Early Onset** **n (%)**	**Late Onset** **n (%)**	**HWE p** **(Early Onset)**	**HWE p** **(Late Onset)**	**OR (95% CI)**	**p**
***NAT2*** **∗** **11A** rs1799929 (481C>T)	Codominant	CC	99 (45%)	20 (50%)	0.3	0.086	Ref.	0.48
		CT	92 (41.8%)	13 (32.5%)			0.69 (0.33-1.48)	
		TT	29 (13.2%)	7 (17.5%)			1.21 (0.47-3.16)	
	Dominant	CC	99 (45%)	20 (50%)			Ref.	0.56
		CT+TT	121 (55%)	20 (50%)			0.82 (0.42-1.60)	
	Recessive	CC+CT	191 (86.8%)	33 (82.5%)			Ref.	0.46
		TT	29 (13.2%)	7 (17.5%)			1.42 (0.57-3.52)	
	Overdominant	CC+TT	128 (58.2%)	27 (67.5%)			Ref.	0.25
		CT	92 (41.8%)	13 (32.5%)			0.66 (0.32-1.36)	
	*Major Allele*	*C*	*290 (0.66)*	*53 (0.66)*			*Ref.*	*0.95*
	*Minor Allele*	*T*	*150 (0.34)*	*27 (0.34)*			*1.02 (0.61-1.68)*	

***NAT2*** **∗** **6B** rs1799930 (590G>A)	Codominant	GG	100 (45.5%)	22 (55%)	0.35	0.027	Ref.	**0.046**
		GA	102 (46.4%)	11 (27.5%)			0.49 (0.23-1.07)	
		AA	18 (8.2%)	7 (17.5%)			1.75 (0.65-4.73)	
	Dominant	GG	100 (45.5%)	22 (55%)			Ref.	0.27
		GA+AA	120 (54.5%)	18 (45%)			0.68 (0.35-1.35)	
	Recessive	GG+GA	202 (91.8%)	33 (82.5%)			Ref.	0.096
		AA	18 (8.2%)	7 (17.5%)			2.34 (0.90-6.10)	
	Overdominant	GG+AA	118 (53.6%)	29 (72.5%)			Ref.	**0.026**
		GA	102 (46.4%)	11 (27.5%)			0.44 (0.21-0.93)	
	*Major Allele*	*G*	*302 (0.69)*	*55 (0.69)*			*Ref.*	*0.98*
	*Minor Allele*	*A*	*138 (0.31)*	*25 (0.31)*			*1.005 (0.60-1.68)*	

***NAT2*** **∗** **7A** rs1799931 (857G>A)	Codominant	GG	202 (91.8%)	36 (90%)	0.33	1	Ref.	0.76
		GA	17 (7.7%)	4 (10%)			1.29 (0.41-4.08)	
		AA	1 (0.4%)	0 (0%)			0.00 (0.00-NA)	
	Dominant	GG	202 (91.8%)	36 (90%)			Ref.	0.74
		GA+AA	18 (8.2%)	4 (10%)			1.22 (0.39-3.83)	
	Recessive	GG+GA	219 (99.5%)	40 (100%)			Ref.	0.55
		AA	1 (0.4%)	0 (0%)			0.00 (0.00-NA)	
	Overdominant	GG+AA	203 (92.3%)	36 (90%)			Ref.	0.66
		GA	17 (7.7%)	4 (10%)			1.30 (0.41-4.10)	
	*Major Allele*	*G*	*495 (95.6)*	*387 (96.8)*			*Ref.*	*0.79*
	*Minor Allele*	*A*	*23 (4.4)*	*13 (3.2)*			*0.86 (0.28-2.59)*	

*∗*SNP: Single nucleotide polymorphism; HWE: Hardy-Weinberg Equilibrium; OR: odds ratio; CI: confidence interval.

*∗*Boldface indicates statistical significance.

**Table 6:** Distribution of expected acetylation phenotypes in early onset group and late onset group.

**rs1799929**	**rs1799930**	**rs1799931**	**Phenotype**	**Early Onset** **n (%)**	**Late Onset** **n (%)**	**Total** **n (%)**	**OR** **(95%CI)**	**p**
CC	GG	GG	Fast	15 (6.82)	3 (7.5)	18 (6.92)	Ref.	
TT			Fast	44 (20)	8 (20)	52 (20)	1.1 (0.26-4.69)	0.89
	GA		Fast	46 (20.91)	6 (15)	52 (20)	1.53 (0.34-6.89)	0.57
		GA	Fast	7 (3.18)	4 (10)	11 (4.23)	0.35 (0.06-2)	0.23
TT			Slow	29 (13.18)	7 (17.5)	36 (13.85)	0.83 (0.18-3.67)	0.8
	AA		Slow	22 (10)	7 (17.5)	29 (11.15)	0.62 (0.13-2.82)	0.54
		AA	Slow	1 (0.45)	-	1 (0.22)	0.67 (0.02-20)	0.82
	AA	GA	Slow	-	-	-	-	-
CT	GA		Slow	44 (20)	5 (12.5)	49 (18.85)	1.76 (0.37-8.26)	0.47
CT		GA	Slow	4 (1.82)	0	4 (1.54)	2.03 (0.08-47.12)	0.67
	GA	GA	Slow	8 (3.64)	0	8 (3.08)	3.83 (0.17-83.44)	0.39

**Total Fast n (%)**				112 (50.9)	21 (52.5)	133 (48.48)	Ref	
**Total Slow n (%)**				108 (49.1)	19 (47.5)	127 (51.52)	0.07 (0.54-2.09)	0.85

*∗*OR: odds ratio; CI: confidence interval.

**Table 7:** Distribution of haplotype frequencies in early onset group and late onset group and association of these haplotypes with onset of psoriasis vulgaris.

**Haplotype**	**Early Onset (f)**	**Late Onset (f)**	**OR (95% CI)**	**p**
TGG	0.3409	0.3375	Ref.	
CAG	0.3136	0.3125	1.00 (0.56-1.78)	0.73
CGG	0.3023	0.3	1.00 (0.55-1.83)	0.51
CGA	0.0432	0.05	1.13 (0.37-3.45)	0.83
TAG	0	0	0	-
CAA	0	0	0	-

*∗*OR: odds ratio; CI: confidence interval.

**Table 8:** The genotype and allele frequencies of *NAT-2* gene polymorphisms in PASI≥12 (severe) group and PASI<12 (mild) group and the relationship of these polymorphisms with severity of psoriasis vulgaris.

**SNP**	**Model**	**Genotype/** **Allele**	**PASI≥12** **(Severe)** **n (%)**	**PASI<12** **(Mild)** **n (%)**	**HWE p** **(PASI≥12)**	**HWE p** **(PASI<12)**	**OR (95% CI)**	**p**
***NAT2*** **∗** **11A** rs1799929 (481C>T)	Codominant	CC	62 (53.5%)	57 (39.6%)	0.07	0.6	Ref.	0.84
		CT	40 (34.5%)	65 (45.1%)			0.57 (0.33-0.97)	
		TT	14 (12.1%)	22 (15.3%)			0.58 (0.27-1.24)	
	Dominant	CC	62 (53.5%)	57 (39.6%)			Ref.	**0.026**
		CT+TT	54 (46.5%)	87 (60.4%)			0.57 (0.35-0.94)	
	Recessive	CC+CT	102 (87.9%)	122 (84.7%)			Ref.	0.43
		TT	14 (12.1%)	22 (15.3%)			0.75 (0.36-1.54)	
	Overdominant	CC+TT	76 (65.5%)	79 (54.9%)			Ref.	0.09
		CT	40 (34.5%)	65 (45.1%)			0.65 (0.39-1.07)	
	*Major Allele*	*C*	*164 (0.71)*	*179 (0.62)*			*Ref.*	***0.04***
	*Minor Allele*	*T*	*68 (0.29)*	*109 (0.38)*			*0.68 (0.47-0.98*)	

***NAT2*** **∗** **6B** Rs1799930 (590G>A)	Codominant	GG	53 (45.7%)	69 (47.9%)	0.84	0.83	Ref.	0.32
		GA	50 (43.1%)	63 (43.8%)			1.02 (0.61-1.71)	
		AA	13 (11.2%)	12 (8.3%)			1.46 (0.61-3.47)	
	Dominant	GG	53 (45.7%)	69 (47.9%)			Ref.	0.2
		GA+AA	63 (54.3%)	75 (52.1%)			1.09 (0.67-1.78)	
	Recessive	GG+GA	103 (88.8%)	132 (91.7%)			Ref.	0.68
		AA	13 (11.2%)	12 (8.3%)			1.44 (0.63-3.32)	
	Overdominant	GG+AA	66 (56.9%)	81 (56.2%)			Ref.	0.13
		GA	50 (43.1%)	63 (43.8%)			0.96 (0.58-1.58)	
	*Major Allele*	*G*	*156 (0.67)*	*201 (0.7)*			*Ref.*	*0.53*
	*Minor Allele*	*A*	*76 (0.33)*	*87 (0.3)*			*1.13(0.78-1.63)*	

***NAT2*** **∗** **7A** Rs1799931 (857G>A)	Codominant	GG	237 (91.2)	187 (93.5)	0.47	0.63	Ref.	0.46
		GA	22 (8.4)	13 (6.5)			0.79 (0.38-1.61)	
		AA	1 (0.4)	0			0.00	
	Dominant	GG	237 (91.2)	187 (93.5)			Ref.	0.43
		GA+AA	23 (8.8)	13 (6.5)			0.75 (037-1.53)	
	Recessive	GG+GA	259 (99.6)	200 (100)			Ref.	0.29
		AA	1 (0.4)	0			0.00	
	Overdominant	GG+AA	238 (91.5)	187 (93.5)			Ref.	0.52
		GA	22 (8.4)	13 (6.5)			0.79 (0.39-1.62)	
	*Major Allele*	*G*	*495 (95.6)*	*387 (96.8)*			*Ref.*	*0.46*
	*Minor Allele*	*A*	*23 (4.4)*	*13 (3.2)*			*1.37 (0.59-3.17)*	

*∗*SNP: single nucleotide polymorphism; HWE: Hardy-Weinberg equilibrium; OR: odds ratio; CI: confidence interval.

*∗*Boldface indicates statistical significance.

**Table 9:** Distribution of expected acetylation phenotypes in PASI≥12 (severe) group and PASI<12 (mild) group.

**rs1799929**	**rs1799930**	**rs1799931**	**Phenotype**	**PASI≥12** **n (%)**	**PASI<12** **n (%)**	**Total** **n (%)**	**OR** **(95%CI)**	**p**
CC	GG	GG	Fast	12 (10.26)	6 (4.2)	18 (6.92)	Ref	
TT			Fast	20 (17.9)	32 (22.38)	52 (20)	0.3125 (0.1-0.96)	**0.04**
	GA		Fast	29 (24.79)	23 (16.08)	52 (20)	0.63 (0.2-1.93)	0.42
		GA	Fast	4 (3.42)	7 (4.9)	11 (4.23)	0.28 (0.06-1.37)	0.12
TT			Slow	14 (11.97)	22 (15.38)	36 (13.85)	0.32 (0.097-1.043)	**0.04**
	AA		Slow	13 (11.11)	16 (11.19)	29 (11.15)	0.4 (0.12-1.38)	0.14
		AA	Slow	1 (0.85)	-	1 (0.22)	1.56 (0.05-43.93)	0.79
	AA	GA	Slow	-	-	-	-	
CT	GA		Slow	18 (15.38)	31 (21.68)	49 (18.85)	0.29 (0.09-0.9)	**0.03**
CT		GA	Slow	2 (1.71)	2 (1.39)	4 (1.54)	0.5 (0.05-4.47)	0.53
	GA	GA	Slow	4 (3.42)	4 (2.8)	8 (3.08)	0.5 (0.09-2.73)	0.42

**Total Fast n (%)**				65 (55.6)	68 (47.6)	133 (48.48)	Ref	
**Total Slow n (%)**				52 (44.4)	75 (52.4)	127 (51.52)	0.73 (0.44-1.18)	0.19

*∗*OR: odds ratio; CI: confidence interval.

*∗*Boldface indicates statistical significance.

**Table 10:** Distribution of haplotype frequencies in PASI≥12 (severe) group and PASI<12 (mild) group and association of these haplotypes with severity of psoriasis vulgaris.

**Haplotype**	**PASI≥12 (f)**	**PASI<12(f)**	**OR (95% CI)**	**p**
TGG	0.2931	0.3785	Ref.	-
CAG	0.3276	0.3021	1.36 (0.89-2.07)	0.16
CGG	0.3276	0.2813	1.46 (0.94-2.28)	0.095
CGA	0.0517	0.0382	1.66 (0.71-3.88)	0.25
TAG	0	0	0	-
CAA	0	0	0	-

*∗* OR: odds ratio; CI: confidence interval.

**Table 11:** * NAT2* allele frequencies in Turkish and other populations.

**Population**	**Detection Method**	**n**	**rs1799929**		**rs1799930**		**rs1799931**		**References**
			**C**	**T**	**G**	**A**	**G**	**A**	
Egyptian	Real-time PCR	199	-	-	0.51	0.49	0.97	0.03	Hamdy et al.2003 [79]
Moroccan	Real-time PCR	163	0.47	0.53	0.75	0.25	0.98	0.02	Guaoua et al. 2016 [80]
Argentinian	Real-time PCR	185	0.63	0.37	0.74	0.26	0.92	0.08	Chamarro et al. 2012 [81]
Brasil	PCR-RFLP	97	0.59	0.41	0.82	0.18	0.96	0.04	Lima dos Santos et al. 2016 [64]
Mexican	Real-time PCR	121	0.68	0.32	0.85	0.15	0.88	0.12	Salazar Gonzales et al. 2014 [82]
Chinese	Ligation detection reaction	686	0.75	0.25	0.77	0.23	0.85	0.15	Wang et al. 2014 [83]
Indian	PCR-RFLP	173	0.68	0.32	0.72	0.28	0.93	0.07	Mishra et al. 2013 [84]
Indonesian	Allele specific PCR	212	0.91	0.09	0.63	0.37	0.85	0.15	Yuliwulandari et al. 2008 [85]
Iranian	PCR-RFLP	229	0.69	0.31	0.62	0.38	0.99	0.01	Torkaman-Bautorabi et al. 2007 [86]
Japanese	Array	225	0.99	0.01	0.82	0.18	-	-	Yoshimura et al. 2003 [87]
Korean	PCR-Sequencing	192	0.98	0.02	0.77	0.23	0.85	0.15	Kang et al. 2009 [88]
Saudi	Real-time PCR	183	0.52	0.48	0.72	0.28	0.89	0.11	Al-Shaqha et al. 2015 [89]
Kazakh	Real-time PCR	320	0.75	0.25	0.75	0.25	0.89	0.11	Iskakova et al.2016 [90]
German	PCR-RFLP	243	0.51	0.49	0.73	0.27	0.98	0.02	Borlak and Reamon-Buetner 2006 [91]
Polish	PCR-RFLP	248	0.62	0.38	0.70	0.30	0.97	0.03	Mrozikiewicz et al. 1996 [92]
Serbs	Allele specific PCR-Taq array	140	0.58	0.42	0.71	0.29	0.99	0.01	Djordjevic et al. 2011 [93]
Turkish	Real-time PCR	104	0.66	0.34	0.77	0.23	0.77	0.23	Tamer et al. 2006 [94]
British	PCR-RFLP	90	0.57	0.43	0.69	0.31	0.98	0.02	Johnson et al. 2004 [95]
Spanish	Allele specific PCR	258	0.53	0.47	0.75	0.25	0.94	0.06	Olivera et al. 2006 [96]
American	Taqman-Allele specific PCR	581	0.54	0.46	0.72	0.28	0.98	0.02	Jiao et al. 2007 [97]
**Turkish**	**Real-time PCR**	**200**	**0.65**	**0.35**	**0,67**	**0.33**	**0.97**	**0.03**	**Current Study**

## Data Availability

The readers may contact the corresponding author for the data underlying the findings of the study.
